# Characterization of the complete chloroplast genome of *Salix chienii* W.C. Cheng (Salicaceae) from Southern China

**DOI:** 10.1080/23802359.2021.1981170

**Published:** 2021-09-27

**Authors:** Weiyu Wang, Xiaoping Li

**Affiliations:** a Collaborative Innovation Center of Southern Modern Forestry, Nanjing Forestry University, Nanjing, China; b College of International Education & College of Forestry, Nanjing Forestry University, Nanjing, China; c Key Laboratory of Forest Tree Genetics and Breeding and High-Efficiency Cultivating in Jiangsu Province, Nanjing Forestry University, Nanjing, China

**Keywords:** *Salix chienii*, *Salix*, chloroplast genome, phylogenetic tree

## Abstract

*Salix chienii* W.C. Cheng (Salicaceae) is a dioecious small tree with high economic and medicinal value. In this study, we reported the complete chloroplast DNA (cpDNA) of *S. chienii* by employing HiSeq sequencing and *de novo* assembly. The chloroplast genome of *S. chienii* is 155,672 bp in length, with a 36.60% GC content; it contains one large single copy (LSC), one small single copy (SSC) and two inverted repeat (IR) regions with lengths of 84,494, 16,330, and 27,424 bp, respectively. A total of 132 genes were predicted, including 85 protein-coding genes, 37 tRNA genes, 8 rRNA genes and 2 pseudogenes. A maximum-likelihood phylogenetic tree showed that *S. chienii* is closely related to *S. wilsonii* and *S. triandra*.

*Salix chienii* W.C. Cheng belongs to the genus *Salix*, family Salicaceae, and is a unique tree species in China (Wang and Fang 1984). As a dioecious species, *S. chienii* can be found as shrubs on both sides of streams at altitudes of 500 to 600 meters in several provinces, including Anhui, Zhejiang, Hunan, Hubei, Jiangsu and Jiangxi. This plant is medically important and can be used for the treatment of fever, sore throat and skin pruritus (Xiao et al. 2004). However, to date, research on *S. chienii* has been limited, and no molecular studies of the species have been conducted. Consequently, the breeding and application of *S. chienii* are restricted, and this plant has not been artificially cultivated.

To promote the protection of *S. chienii* germplasm resources, we reported the complete chloroplast genome sequence of *S. chienii* (GenBank accession number: MW969692) based on Illumina paired-end sequencing in this study. We also determined its phylogenetic relationship with other species of the Salicaceae family to better understand their interrelation.

Fresh, healthy leaves of *S. chienii* were collected from Mount Huangshan (Anhui, China; coordinates: 118°17′25.75″E, 30°11′22.68″N), and the voucher specimen was deposited in Room 60708, Biotechnology Building, Nanjing Forestry University, China (Xiaoping Li, xpli@njfu.edu.cn, HSYYL2020001). Total genomic DNA was extracted from the fresh leaves using the DNeasy Plant Mini Kit (Qiagen, Valencia, CA, USA) and was further used to construct an Illumina paired-end (PE) genome library. Whole-genome sequencing was conducted with 150 bp paired-end reads on the Illumina NovaSeq 6000 Platform (Illumina, USA) (Genepioneer Biotechnologies Co. Ltd, Nanjing, Jiangsu, China). Approximately 13.4 G of raw reads were generated after sequencing. Low-quality reads were trimmed by using Fastp (Chen et al. 2018). The clean reads were assembled using the software NOVOPlast (Dierckxsens et al. 2017) and then annotated and corrected by using PGA (Plastid Genome Annotator) and Geneious Prime version 2021.1.1 (Qu et al. 2019; Kearse et al. 2012) through comparison with the complete chloroplast genome of its genetically close species *S. babylonica* (MF189169.1; Wang and Yang 2016).

The chloroplast genome of *S. chienii* was 155,672 bp in length and contained a small single-copy region (SSC; 16,330 bp), a large single-copy region (LSC; 84,494 bp) and a pair of inverted repeat regions (IR; 27,424 bp). The total GC content of the chloroplast genome was 36.6%, with different values for the SSC (31.0%), LSC (34.4%), and IR (41.7%) regions. A total of 132 genes were annotated from the chloroplast genome sequence, including 85 protein-coding genes, 37 transfer RNA (tRNA) genes, 8 ribosomal RAN (rRNA) genes and 2 pseudogenes. A total of 19 genes, including eight protein-coding genes, seven tRNAs and four rRNAs, were duplicated in the IR regions.

To explore the phylogenetic position of *S. chienii* among *Salix*, a phylogenetic analysis was performed by comparing the chloroplast genome sequences of *S. chienii* with 21 other Salicaceae species. All sequences were downloaded from the GenBank database and aligned by MAFFT v7 (Katoh and Standley 2013), and a phylogenetic tree was constructed by using IQ-TREE v2.1.3 (Nguyen et al. 2015) based on the maximum likelihood (ML) algorithm (with the best model of K3Pu + F+R8 and 1000 bootstrap replicates). In order to add a distinct root to the ML tree, *Hevea brasiliensis* (Tangphatsornruang et al. 2011) derived from Euphorbiaceae was set as an outgroup. The bootstrap support value (%) is shown next to the nodes. Our ML phylogenetic tree showed that *S. chienii* was closely related to the two congeners, *S. wilsonii* and *S. triandra*, with high bootstrap values (Figure 1). The complete chloroplast genome of *S. chienii* presented here may contribute to the further study of genetic diversity, molecular identification and phylogenetic classification of the genus *Salix* (Salicaceae).

**Figure 1.**
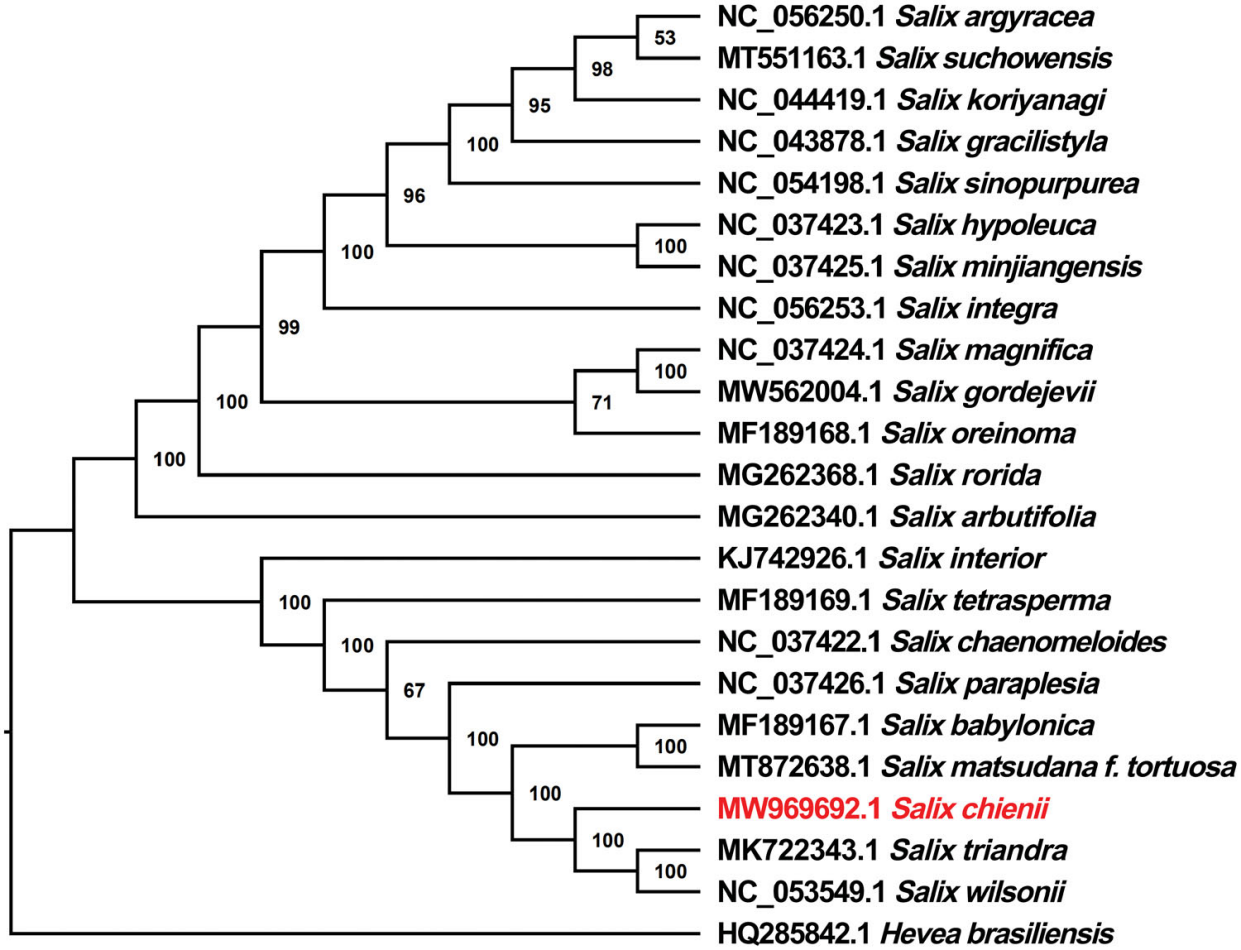
A maximum likelihood phylogenetic tree of *S. chienii* and 21 other Salicaceae species based on chloroplast genome sequences; *Hevea brasiliensis* was used as an outgroup. Bootstrap support values are indicated for each node.

## Data Availability

The sequence data that support the findings of this study are openly available in GenBack of NCBI https://www.ncbi.nlm.nih.gov/ under the accession no.MW969692. The associated BioProject, Bio-Sample and SRA numbers are PRJNA727759, SAMN19036906 and SRR14479090, respectively.
